# The amygdala and the relevance detection theory of autism: an evolutionary perspective

**DOI:** 10.3389/fnhum.2013.00894

**Published:** 2013-12-30

**Authors:** Tiziana Zalla, Marco Sperduti

**Affiliations:** ^1^Institut Jean Nicod, Centre National de la Recherche Scientifique, Ecole Normale SupérieureParis, France; ^2^Laboratoire Mémoire et Cognition, Institut de Psychologie, Université Paris DescartesBoulogne-Billancourt, France; ^3^Inserm U894, Centre de Psychiatrie et Neurosciences, Université Paris DescartesParis, France

**Keywords:** autism spectrum disorders, amygdala, ventromedial prefrontal cortex, self-relevance, social brain

## Abstract

In the last few decades there has been increasing interest in the role of the amygdala in psychiatric disorders and, in particular, in its contribution to the socio-emotional impairments in autism spectrum disorders (ASDs). Given that the amygdala is a component structure of the “social brain,” several theoretical explanations compatible with amygdala dysfunction have been proposed to account for socio-emotional impairments in ASDs, including abnormal eye contact, gaze monitoring, face processing, mental state understanding, and empathy. Nevertheless, many theoretical accounts, based on the *Amygdala Theory of Autism*, fail to elucidate the complex pattern of impairments observed in this population, which extends beyond the social domain. As posited by the *Relevance Detector* theory (Sander et al., 2003), the human amygdala is a critical component of a brain circuit involved in the appraisal of self-relevant events that include, but are not restricted to, social stimuli. Here, we propose that the behavioral and social–emotional features of ASDs may be better understood in terms of a disruption in a “Relevance Detector Network” affecting the processing of stimuli that are relevant for the organism’s self-regulating functions. In the present review, we will first summarize the main literature supporting the involvement of the amygdala in socio-emotional disturbances in ASDs. Next, we will present a revised version of the *Amygdala Relevance Detector* hypothesis and we will show that this theoretical framework can provide a better understanding of the heterogeneity of the impairments and symptomatology of ASDs. Finally, we will discuss some predictions of our model, and suggest new directions in the investigation of the role of the amygdala within the more generally disrupted cortical connectivity framework as a model of neural organization of the autistic brain.

## INTRODUCTION

Autism spectrum disorders (ASDs) are pervasive developmental disorders characterized by a triad of symptoms including abnormal socio-emotional processing, verbal and non-verbal communication problems, and restricted interests and repetitive behaviors (American Psychiatric Association, 2000). Although there is now substantial evidence implicating genetic bases and brain mechanisms in ASD etiopathology (see Eapen, 2011), there is no apparent core neurocognitive dysfunction associated with a single structure that could esaustively explain the variety of symptoms observed in these disorders. Current data rather suggest that multiple perceptual and cognitive processes subserved by different neural systems are affected. However, it is possible that the dysfunction of a single structure of an interconnected neural circuit, such as a circumscribed damage to the amygdala, can influence other areas of the circuit and have widespread repercussions on multiple cognitive functions, especially if this occurs early in development (Bachevalier, 2005).

Several theories have been proposed to account for the atypical pattern of socio-emotional behavior in ASDs. The most influential are the *Theory of Mind* (Baron-Cohen et al., 1985; Baron-Cohen, 1995), the *Socio-emotional* theory (Hobson, 1993), the *Social Motivation* theory (Grelotti et al., 2002; Schultz et al., 2003; Schultz, 2005), and the *Fast-track modulator* model (Senju and Johnson, 2009). While a full description of these theories is beyond the scope of the present paper, and we direct the interested reader to two recent extensive reviews (Gaigg, 2012; Hamilton, 2013), what we intend to emphasize here is that all these proposals are compatible with a core deficit of the so-called “social brain” in which the amygdala is the key component.

Alternatively, in the present review, we will acknowledge that the function of the human amygdala is better characterized in terms of a *self-relevance detection* system (Sander et al., 2003) and, based on theoretical argument and experimental support taken from cognitive neuroscience and evolutionary biology, we argue that abnormalities in this structure associated with the disruption of this self-relevance detection system would potentially explain a larger variety of impairments and symptomatology of ASDs that include, but are not restricted to, the social domain. In this view, the amygdala, originally designed to automatically detect potentially threatening or dangerous environmental events under ancestral conditions, has enlarged its domain of specificity in humans to respond to a broader range of self-relevant information in the physical and social environment, including intrinsic biological features and extrinsic context-dependant information. As previously defined (Sander et al., 2003), an event is relevant for an organism if it significantly influences (positively or negatively) the attainment of his or her goals, the satisfaction of his or her needs, the maintenance of his or her well-being within the physical environment and the social context. Following the theoretical account advocated by the *Relevance Detection Theory of the Amygdala* (Sander et al., 2003), the present proposal aims to specify the role of the amygdala in the ASD etiopathology by highlighting the notion that this structure is a key component of a larger interconnected fronto-limbic neural system. Because of its complex functional connectivity with the ventromedial prefrontal cortex (vMPFC), a stimulus is deemed relevant through two distinct processes of salience attribution: (a) the intrinsic salience of a stimulus, which is determined by its biologically innate (e.g., threat, food) or physical (e.g., intensity or novelty) features, via stimulus-driven bottom-up low level processes, and (b) the extrinsic or *context-dependent* salience which can be assigned flexibly through top-down evaluative processes.

In the present work, we will first discuss the early formulation of the “amygdala theory” of autism (Baron-Cohen et al., 2000), and review research on amygdala function in subjects with and without ASDs that calls into question the specific social view as stated by the “hard” formulation of the “amygdala theory.” We will then propose a more general view based on the notion that the amygdala is a critical component of a brain circuit responsible for the detection of relevant stimuli or events, and crucially for the formation of a “salience map” that integrates and prioritizes salience signals from various sources of information, in accordance with the motivations and the contextual goals of the perceiver.

## THE ”AMYGDALA THEORY OF AUTISM”

Based on research on animal lesion (Kling and Brothers, 1992), single cell recording studies (Brothers et al., 1990) and neurological studies, Brothers (1990) has proposed that a brain network including three regions, the amygdala, the orbitofrontal cortex (OFC), and the superior temporal gyrus (STG), constitutes the neural basis of social intelligence, the so called “social brain.” Given that social perception impairment, abnormal gaze behavior and emotional processing are central to the autistic symptomatology, it is not surprising that a great emphasis has been placed on amygdala involvement in the etiopathology of this condition. Nevertheless, the exact role of this structure in the behavioral deficit of ASDs, above all in the social domain, is still a controversial issue.

Baron-Cohen et al. (2000) posited that damage or dysfunction of the amygdala should be at the root of social impairments in ASDs and proposed the *Amygdala Theory of Autism*. The “hard” formulation of this theory states that “…the amygdala is one of several neural regions that are *necessarily* abnormal in autism” (Baron-Cohen et al., 2000, p. 1; emphasis added). To sustain this claim, the authors presented converging evidence coming from animal models, post-mortem and structural studies showing abnormalities in the amygdala in ASDs, as well as behavioral similarity between subjects with ASDs and patients with amygdalotomy. Furthermore, the authors reported a fMRI study on adults with high functioning autism (HFA) and Asperger syndrome (AS) showing difficulties in identifying mental state/emotional information from the eyes of others (reading the mind in the Eyes task) that was associated with weaker amygdala activation, as compared to typically developing subjects. The reduced amygdala response to the intentional meaning of the emotional expressions in adults with ASDs is consistent with a large amount of studies reporting atypicalities in face processing in infants in the first 6 months of life (Maestro et al., 2002) and abnormal fixation to the eye region in adults (Klin et al., 2002; Pelphrey et al., 2002; Adolphs et al., 2005; Dalton et al., 2005).

However, evidence from animal research seems to challenge this hypothesis. In a series of studies, Emery et al. (2001) used the rhesus monkey as a model system to examine the role of the amygdala in conspecific social behavior, and showed that, in dyadic social interactions, adult monkeys with extensive bilateral lesions of the amygdala can decode and generate social gestures and initiate and receive more affiliative social interactions than control monkeys. Importantly, the monkeys exhibited abnormal response to normally fear-inducing stimuli such as snakes, and the normal reluctance to engage with a novel animal was eliminated. Reduced fear response and socially uninhibited behavior were also observed in primates at 2 weeks of age with bilateral lesions of the amygdala (Prather et al., 2001).

More recently, the specific role of the amygdala in social cognition in ASDs has been questioned in a study of two rare patient cases suffering from Urbach–Wiethe disease, which is characterized by a developmental selective atrophy of the bilateral amygdala (Paul et al., 2010). In fact, even if these patients reported some social deficit associated with ASD symptomatology, their overall performance on the standard diagnostic test for ASDs and in clinical examination did not reveal a clear association with ASD symptomatology. A direct evidence comes from a recent study by Birmingham et al. (2011) showing that the differences between individuals with amygdala lesions and ASDs are more striking than the similarities. Indeed, while patients with amygdala damage failed to attend to social features in stimulus-driven manner, but showed an intact modulation of eye gaze by the task, the ASD group exhibited a notable absence of such task-dependent modulation. The authors concluded that social disturbance in ASDs would be better understood in terms of a disruption of the complex network of structures with which the amygdala is connected rather than in the amygdala itself.

Because of its widespread functional connections with sensory, associative areas and autonomic systems, the amygdala is regarded as a “sensory gateway” and plays an important role in the integration of a wide array of visceral, sensory, and cognitive information (Freese and Amaral, 2009). The fact that the amygdala receives projections from both subcortical and cortical pathways confirms the view that multiple processes may be engaged depending on the type of information involved, but of particular interest for the present proposal are the functional connections with the vMPFC which relay amygdala input to regions involved in more deliberate forms of decision making reasoning and cognitive control. It is, in fact, well documented that the amygdala has multiple connections to prefrontal areas, receiving from and relaying information to areas of insular, OFC, and lateral prefrontal cortex (Stefanacci and Amaral, 2000). These reciprocal connections extend the functionality of the amygdaloid structure which is responsive to the entire state of the organism and contextual information (Mosher et al., 2010).

In a recent review, Gaigg (2012) discusses results from studies on emotional arousal, aversive conditioning, and reward contingency learning in ASDs and concludes that the results are globally inconsistent with the view that only emotions relevant to social cognition are compromised in ASDs. Noteworthy, the author emphasizes that theories uniquely based on a dysfunctional social brain network ignore multiple aspects of the interpersonal emotional disturbance and the more widespread anomalies in the general domain of emotions in ASDs. Overall, current findings in subjects with and without ASDs challenge the “hard” formulation of the *Amygdala Theory of Autism*, primarily grounded on the *social function* view of the amygdala, and question its role in ASD symptomatology.

## THE AMYGDALA: ANATOMICAL AND NEUROIMAGING FINDINGS IN TYPICAL DEVELOPMENT

The amygdala is an almond-shaped group of subcortical nuclei belonging to the limbic system situated in the deep medial temporal lobe. Even though, in most neuroimaging studies, it is considered as a whole, the amygdala is composed of several subnuclei that present specific cytoarchitectonic features and different patterns of connectivity with several subcortical and cortical structures. The amygdala has been divided into three major subdivisions^1^: the laterobasal, the centromedial, and the superficial nuclei, each of them being associated with a specific coactivation profile (Bzdok et al., 2012).

While anatomical connectivity of the amygdala has been largely elucidated by non-human primate studies (Amaral and Price, 1984; Barbas and De Olmos, 1990), with the advent of non-invasive neuroimaging techniques, an increasing number of studies have been devoted to determining the functional connectivity of the human amygdala. The amygdala does not work in isolation, but rather serves as a complex node within multiple neural networks (Pessoa, 2008). Using a connectivity-based parcellation approach, Bzdok et al. (2012) identified three distinct clusters in human amygdala based on their brain-wide coactivation maps. These analyses revealed that the laterobasal nuclei group of the amygdala is linked with the integration of high-level sensory inputs (visual, auditory, gustatory, somatosensory, and, in part, olfactory environmental information), and the representation of stimulus-value associations. Its centromedial nuclei group is, in turn, functionally associated with attentional, vegetative, and motor responses, while the superficial nuclei group is found to process olfactory information.

Research in humans (Roy et al., 2009; Robinson et al., 2010) is fundamentally in agreement with studies in macaque monkeys showing widespread connections of the amygdala with cortical and subcortical regions encompassing the anterior cingulate cortex (ACC) and the inferior and medial prefrontal cortex, the hippocampus and the parahippocampal gyrus, the temporal lobe and the insula (Amaral and Price, 1984; Barbas and De Olmos, 1990; Stefanacci et al., 1996; Ghashghaei and Barbas, 2002). Using probabilistic diffusion tensor parcellation, Bach et al. (2011) have shown that the superficial portion, approximately corresponding to the centromedial and the superficial nuclei, and the deep portion, corresponding to the basal nucleus, are preferentially connected with OFC and the temporal pole, respectively. Recently, using resting state data Mishra et al. (2013) replicated this pattern of connectivity and additionally reported that the superficial nucleus shows greater connectivity with motor and MPFC regions, while the deep nucleus is strongly functionally coupled with the middle frontal gyrus and inferior parietal lobe.

Functional neuroimaging studies have demonstrated that the amygdala is implicated in a large variety of cognitive and behavioral functions, including fear conditioning (Adolphs et al., 2005), memory formation (Packard and Cahill, 2001), learning of stimulus–reward associations (Baxter and Murray, 2002), social and affective processing (Anderson and Phelps, 2000; Hariri et al., 2002), appraisal of positive (winning) and negative (losing) emotions elicited during a competitive contest (Zalla et al., 2000), as well as in a multiplicity of high-order cognitive functions ranging from emotional control (Ochsner et al., 2004; Goldin et al., 2008) to self-awareness and social perception. In the domain of social cognition, a large variety of stimuli and situations are associated with amygdala activation in typical development, including gaze direction (Kawashima et al., 1999; George et al., 2001; Wicker et al., 2003), eye contact (Emery, 2000), face identity, trustworthiness (Adolphs et al., 1998), facial familiarity (Dubois et al., 1999), racial outgroup faces (Hart et al., 2000; Phelps et al., 2000), body movements (Bonda et al., 1996), attribution of others’ mental states and communicative intents (Baron-Cohen et al., 1999; Hart et al., 2000; Portas et al., 2000).

Alternative views have emphasized the role of the amygdala as a mechanism for more general vigilance and attention orientation (Davis and Whalen, 2001). Along this line, Vuilleumier (2005) showed that, while normal subjects exhibited enhanced brain activity in visual areas for fearful faces, patients with amygdala lesions did not show the same effect, suggesting that the role of the amygdala is to modulate the processing of sensory input that might be relevant for its vital significance, both directly and by top-down signals. This function has also been demonstrated using tasks in which emotional information is prioritized and receives privileged access to consciousness and attentional resources (Vuilleumier and Schwartz, 2001).

Remarkably, although the amygdala is involved in processing a wide range of emotions, comprising positive ones (Costafreda et al., 2008; Sergerie et al., 2008; Bzdok et al., 2012), it has been suggested that it specifically responds to the degree of arousal and not to the valence of the stimulus (Small et al., 2003; Costafreda et al., 2008), and studies that have independently manipulated valence and intensity have provided evidence that amygdala activity is preferentially involved in processing the affective intensity rather than the valence of the event (Anderson et al., 2003; Small et al., 2003). Interestingly, Cunningham et al. (2008) showed that, in concert with other neural components of evaluative processing, the amygdala may respond flexibly to the valence and intensity of stimuli in goal-congruent fashion, although it processes negativity in a less flexible fashion than positivity.

## THE AMYGDALA: ANATOMICAL AND NEUROIMAGING FINDINGS IN ASDs

A seminal post-mortem study on ASDs children reported abnormal cell packing primarily in the medial temporal lobe regions comprising the hippocampus and the amygdala (Bauman and Kemper, 1985). Preliminary volumetric *in vivo* studies of amygdala morphology in ASDs have reported contrasting evidence showing either increased (Abell et al., 1999; Howard et al., 2000), decreased (Aylward et al., 1999), or no difference (Haznedar et al., 2000) in amygdala volume. These contrasting results could be explained by several factors, such as the heterogeneity of the studied sample with respect to psychometric (e.g., IQ) and demographic (e.g., age) measures, differences in data analysis, or the small number of subjects in single studies. Recently, neuroimaging meta-analytic techniques have allowed researchers to partially overcome these limitations, allowing the pooling of large datasets.

In a meta-analysis of 19 voxel-based morphometry (VBM) studies, Duerden et al. (2012) reported a decrease in right amygdala volume in child/adolescent subjects with ASDs, but not in adults. However, more extensive meta-analyses have consistently reported volume decrease in the amygdala, particularly in the right hemisphere, even in adults with ASDs (Cauda et al., 2011; Via et al., 2011). In a more recent meta-analysis, Nickl-Jockschat et al. (2012) reported a significant decrease of gray matter volume in a cluster in the medial temporal lobe that did not include the amygdala. This is probably due to the fact that in the latter study authors used probabilistic cytoarchitectonic maps to localize anatomical regions corresponding to significant clusters of decreased gray matter volume. This approach is much more reliable, especially for structures whose anatomical borders are not as easily determinable as those of the amygdala, allowing assignment of cluster sites to histologically defined brain regions in a probabilistic fashion. Indeed, using the same localization approach, Ball et al. (2009) showed that only about 50% of peaks reported as amygdala activation across 114 functional neuroimaging studies could reliably be assigned with a probability ≥80% to this structure. A summary of the meta-analytic results described above is presented in **Table 1**.

**Table 1:** Summary of meta-analytic VBM results in the amygdala.

Study	Number of studies included	Total number of subjects (ASDs/TD)	Age ASDs (Ch–Ado/Adu)	Age TD (Ch–Ado/Adu)	Results (Ch–Ado/Adu)
Duerden et al. (2011)	19	253/289 (Ch–Ado) – 70/80 (Adu)	11.4 ± 3/33.4 ± 4.3	11.5 ± 2.7/32.4 ± 5.2	< R Amy/=
Cauda et al. (2011)	16	350/368	18.5 ± 8.2	18.1 ± 7.7	< R Amy
Via et al. (2011)	20	272/243 (Ch–Ado) – 224/228 (Adu)	13 ± 4.6/28 ± 10.1	12 ± 4.3/27 ± 9.7	< L–R Amy/< L–R Amy
Nickl-Jockschat et al. (2012)	16	277/303	18.5 ± 9.9	18.2 ± 9.6	=

*Results for meta-analytic contrasts comparing gray matter volume between TD and ASDs. When available, results are separately reported for Ch–Ado and Adu. Only results concerning the amygdala are reported. Ch–Ado, children–adolescents; Adu, adults; Amy, amygdala; R, right; L, left; <, decreased gray matter volume in ASDs; =, no difference between groups.*

It is noteworthy that, in comparison with studies reporting gray matter changes, there are few studies investigating the anatomical connection between the amygdala and other cortical–subcortical structures in ASDs. Preliminary diffusion tensor imaging (DTI) studies have reported, among other structures, altered fractional anisotropy (FA), a measure of fiber tracts integrity, in regions surrounding the amygdala (Barnea-Goraly et al., 2004; Noriuchi et al., 2010) or in tracts connecting the amygdala and the fusiform gyrus (Conturo et al., 2008). Other studies have shown reduced FA in specific fiber tracts, such as the inferior longitudinal fasciculus and inferior fronto-occipital fasciculus connecting the amygdala, the fusiform face area (FFA), and the superior temporal sulcus (STS; Jou et al., 2011), and in the uncinate fasciculus connecting the lateral and medial OFC with the anterior portion of the temporal lobe, including the amygdala (Radua et al., 2011).

In functional neuroimaging research, the involvement of the amygdala in the physiopathology of ASDs has been advocated either in terms of *hypoactivation* or *hyperactivation* of this structure. According to the “hypo-active models,” the amygdala fails to process social stimuli as meaningful with the result that they do not receive preferential attention (Schultz, 2005), while in “hyper-active models,” social stimuli are thought to cause an aversive over-arousal, with the result that they are actively avoided (Dalton et al., 2005; Corden et al., 2008). Structural and functional studies in ASD subjects failed, however, to report a systematic hypo- or hyperactivation of the amygdala. Baron-Cohen et al. (2000) found diminished amygdala activity in ASD subjects, but there is convincing evidence of amygdala hyperactivity in adults with ASDs when they gaze at the eye region (Dalton et al., 2005; Kliemann et al., 2012). In a more recent study by von dem Hagen et al. (2013), control subjects showed increased activation in the amygdala when contrasting neutral faces with direct *vs* averted gaze, while ASD participants showed an inverse pattern of activation.

Indirect behavioral evidence of amygdala functions in people with ASD are provided by fear conditioning protocols. For example, Gaigg and Bowler (2007) reported a pattern of abnormalities in differential fear conditioning in individuals with ASDs. In contrast, Hall et al. (2010) did not find any difference in brain activity between ASDs and control participants when presenting sub-threshold anxious expressions and, similarly, South et al. (2011) showed preserved fear acquisition in individuals with ASDs using aversive conditioning. Overall, these findings suggest that amygdala reactivity in ASDs is not absent, but response variability may depend on several factors, such as fixation to eye region, gaze avoidance and, as we argue in this review, more crucially on the abnormal fronto-amygdala connectivity associated with the diminished modulatory role of the vMPFC on this structure.

There is a growing consensus that the cognitive and behavioral disturbance in ASDs cannot be fully understood in terms of local dysfunction but are better viewed as impairments of functional networks (Kana et al., 2011). The fronto-amygdala disconnectivity explanation is consistent with a more general disrupted cortical connectivity framework (Belmonte et al., 2004), as a model of ASDs neural organization (Just et al., 2004; Geschwind and Levitt, 2007). Reduced activity of a fronto-parietal network was associated with a task requiring the flexible allocation of cognitive resources to guide goal-directed behavior in participants with ASDs (Solomon et al., 2009). Monk et al. (2010) reported altered connectivity between the right amygdala, subgenual vMPFC and middle temporal gyrus during emotional face processing, and diminished top-down modulation has been reported in studies using face processing and imitation (Cook et al., 2012). Disrupted connectivity between the OFC and the amygdala is supported by resting state data showing altered long-range connectivity in ASDs participants (Cherkassky et al., 2006; Anderson et al., 2011a, b) at both the structural and functional levels (Radua et al., 2011; Swartz et al., 2013).

Interestingly, Kleinhans et al. (2008) have shown that altered functional connectivity between the amygdala and the FFA during a face identification task correlates with the severity of social impairment in adults with HFA. In a subsequent study, Kleinhans et al. (2009) observed diminished amygdala habituation in response to neutral faces in subjects with ASDs, compared to subjects with typical development, and lower level of habituation correlated with the amount of social impairment. In accordance with these results, Swartz et al. (2013) showed that reduction of amygdala habituation to neutral and sad faces correlates with symptom severity, and that connectivity between the vMPFC and the amygdala was reduced in young subjects with ASDs. Overall, these data suggest that amygdala habituation correlates with symptom severity, and, that both phenomena could reflect the disrupted connectivity between the amygdala and the MPFC.

The idea of a key role of a single structure, the amygdala, seems difficult to reconcile with the view that the neuropathology of autism involves impaired widespread connectivity throughout the brain. However, as revealed by a recent study by Gotts et al. (2012), disrupted connectivity in high-functioning adolescents with ASDs, relative to typically developing adolescents, is most pronounced between limbic-related brain areas involved in affective processing, particularly in the amygdala and the vMPFC. More importantly, it has been shown that early damage to medial temporal lobe structures, including the amygdala, has widespread repercussions on other neural systems, such as a delayed maturation of the dorsolateral prefrontal cortex (Bertolino et al., 1997) and a dysregulation of striatal dopaminergic neurotransmission (Saunders et al., 1998). This view suggests that early developmental dysfunction in the medial temporal lobe (amygdala, hippocampus, and parahippocampus) in ASDs may cause a breakdown in brain connectivity that are normally recruited during complex cognitive tasks and trigger abnormal development of the prefrontal cortex (Dawson et al., 2002).

## THE RELEVANCE DETECTION THEORY OF THE AMYGDALA

The amygdala is a structure of the mammalian limbic system, shaped by evolution to rapidly and automatically detect potentially threatening or dangerous environmental events, and for learning about contingencies that are likely to predict similar events in the future (Amaral and Price, 1984; Öhman and Mineka, 2001; LeDoux, 2005). In virtue of its primary adaptive function, threatening or dangerous events are detected automatically and rapidly through the physiological mechanism of emotional arousal (Lang et al., 1993; Critchley et al., 2002). Emotional arousal allows recruiting additional cognitive and attentional resource allocation (Anderson and Sobel, 2003), facilitating access to awareness (Vuilleumier and Schwartz, 2001) and enhancing encoding and memory through an automatic process mediated by the sub-cortical amygdalar–hippocampal route (Kensinger and Corkin, 2004). Other findings indicate that the amygdala is important in the implicit processing of emotional stimuli. The inducing of amygdala responses by pre-attentively processed faces expressing threat (Vuilleumier et al., 2003) and fearful or happy faces (Juruena et al., 2010) presented by backward masking is thought to reflect the functioning of a primitive pathway specifically devoted to the rapid unconscious processing of socio-emotional events encompassing explicit cognitive assessments (Sergent, 1994).

As posited by the “Relevance Detection Theory” (Sander et al., 2003), the human amygdala is a component of an extended neural cortico-limbic system involved in detecting stimuli by focusing attentional and physiological resources on cues that have special relevance for the safety or success of an organism within the broader context of its social life. As previously defined (Sander et al., 2003, p. 311), “*An event is relevant for an organism if can significantly influence (positively or negatively) the attainment of his or her goals, the satisfaction of his or her needs, the maintenance of his or her well-being, and the well-being of his or her species. According to this view, fearful and angry faces represent relevant information because they potentially obstruct one’s goal and signal the presence of a danger for the organism and his or her con-specifics*.”

From a phylogenetic perspective, in the primitive mammalian brain, the amygdala is part of a modular system shaped by evolution to detect potentially threatening physical events and biological stimuli (e.g., spiders, snakes), and to prepare the organism for action by facilitating escape and avoidance (LeDoux, 1996). MacLean (1970) provided an evolutionary explanation of emotion and social intelligence. In particular, he proposed that emotions engage relatively primitive circuits that are conserved throughout mammalian evolution, along with the idea that structures in neocortex are specialized in cognitive and deliberative processing, such as action planning, decision making, and social cognition. Originally designed to signal potential threat and danger under ancestral conditions, the human amygdala has evolved, conjointly with the cortical structures, to serve to alert an organism toward a broader range of self-relevant information, including appetitive and aversive events coming from the internal milieu as well as from the physical and social environment to promote more adaptive behavior and flexible social exchanges. More crucially, it responds flexibly to stimuli whose relevance is contextually and cognitively modulated and is associated with various affective experiences (Cunningham et al., 2008).

Cross-species comparative studies have provided evidence of the co-evolution of the amygdaloid complex and the prefrontal areas in the neocortex (Barton and Aggleton, 2000) substantiating the view that this structure is a critical component of the integrative cortico-limbic network that constitutes an unitary evolved system for the detection of relevant events (Sander et al., 2003). The amygdala is involved in enhancing sensory processing and orienting visuo-spatial attentional resources toward salient features of the stimulus through both direct (amygdala–visual cortex) and indirect (amygdala–prefrontal cortex–visual cortex) connections, while the “quick-and-dirty” response relies on the activation of the arousal systems via the direct sub-cortical afferent route from all sensory modalities and the efferent connections with hypothalamic and brain-stem nuclei (LeDoux, 1995).

With respect to these distinct cortical pathways, one might distinguish the *intrinsic* and the *extrinsic* types of salience. While certain stimuli are intrinsically (or innately) self-relevant, because of their biological significance (e.g., threat, food, anger) or physical features (e.g., loudness, brightness, intensity, frequency of appearance, etc.), the extrinsic salience is flexibly acquired through context-dependant and conscious appraisal processes. Thus, the computational role of the human amygdala is twofold: on the one hand, it automatically and rapidly detects physically and biologically relevant information, via bottom-up processes, reflecting its more primary function; on the other hand, it integrates multiple salience signals originated via a top-down processes so as to create a priority map of intrinsically and extrinsically self-relevant information. Importantly, while the amygdala is specifically responsible for processing stimulus or event salience, which is a more fundamental feature since it is a measure of its importance, in a strict biological sense, value signals coding positivity for appetitive stimuli and negativity for aversive stimuli (Navalpakkam et al., 2010) are dynamically construed in vMPFC (Harris et al., 2011).

Direct evidence for this theory in humans is provided by neuroimaging studies. For example, food stimuli are more salient if we are hungry (LaBar et al., 2001) and very intense stimuli can lose their salience if they are repetitive, as shown by the habituation phenomenon (Marks and Tobeña, 1991). Morris and Dolan (2001) observed that amygdala activation was positively correlated with recognition memory scores for food items and that participants showed enhanced recognition of food stimuli (relative to non-food) in a fasting state. This enhanced recognition for food stimuli was significantly reduced when participants were in a satiated state. In accordance with this idea, Mohanty et al. (2008) investigated the neural mechanisms underlying attention toward food in participants when hungry and satiated, varying the relevance of the food stimuli. When hungry, participants showed increased amygdala activation to pictures of food and faster attentional orienting toward food cues as well as increased connectivity between limbic areas and parietal attention regions subserving attentional shifts, compared to when they were satiated.

Ousdal et al. (2008) reported increased amygdala activation toward letter stimuli, which are non-emotional and non-social, when the letters were targets in a go/no-go task and thus behaviorally relevant to participants’ performance with respect to one’s ongoing motivational state. In a further study using neutral task-dependant stimuli, Ousdal et al. (2012) suggested that when the relevance of a stimulus is determined by a specific task or context, the amygdala activity is modulated by cortical activity in the prefrontal cortex, based on context or prior knowledge.

Notably, results about people with ASD’ performances in go/no-go tasks are mixed, with some studies reporting impaired performances (Ozonoff et al., 1994; Langen et al., 2012; Xiao et al., 2012) or only subtle difference (Geurts et al., 2009), while others showed comparable performances in this task (Happé et al., 2006; Schmitz et al., 2006; Lee et al., 2009). Xiao et al. (2012) showed that impairment in the go/no-go task was associated with decreased right prefrontal cortex activity during no-go blocks, while in Schmitz et al.’s (2006) study, increased prefrontal activity was found in ASD group for correct inhibited no-go trials. These results seem to suggest that in general prefrontal dysfunction is related to diminished performance in the go/no-go task, but that compensatory mechanism could be observed and lead to comparable performance thus explaining the contrasting behavioral results. The direct link between these studies in ASDs and that of Ousdal et al. (2012) is not straightforward since in classical go/no-go tasks the behavioral relevance of the stimulus (no-go) is not manipulated independently of its frequency. Thus, in this case, it is not easy to disentangle the role of frequency (that in our framework could be considered as “intrinsic salience”) and behavioral salience (that in our framework could be considered as “extrinsic salience”). Overall, this handful of studies evidence the possibility that salient stimuli in these protocols (the no-go trials) are processed, at least in some cases, less efficiently in participants with ASD and that this might be linked to prefrontal cortex dysfunctions.

The amygdala also appears to be important in stimulus–reward association (Schoenbaum et al., 1998) or when magnitude of reinforcement needs to be maintained in working memory in order to accomplish a successful performance (Kesner and Williams, 1995), in processing positive words (Hamann and Mao, 2002), positive pictures (Hamann et al., 1999, 2002; Canli et al., 2001; Garavan et al., 2001), pleasant tastes (O’Doherty et al., 2001), or expectation of pleasant tastes (O’Doherty et al., 2002).

In the socio-emotional domain, N’Diaye et al. (2009) have showed that amygdala response to facial emotion is modulated by interaction between the expressed emotion and gaze direction: greater activation has been reported for fearful faces with averted gaze, signaling a possible threat, and for anger expression with direct gaze, signaling aggression. Similarly, increased activation in the amygdala was observed when contrasting neutral faces with direct *vs* averted gaze in control subjects indicating that an angry face is more *relevant* if the gaze is directed at the observer than if it is averted (von dem Hagen et al., 2013).

The role of the amygdala as a relevance detector is also consistent with neurophysiological findings in non-human primates showing that the neural response in this structure codes not only the raw value of a stimulus, i.e., the negative or positive representation of a stimulus, but also its “state value” (Morrison and Salzman, 2010). The latter takes into account the internal (e.g., hunger) and external (e.g., a specific rule) parameters of a given situation. One study also reported that amygdala is responsive to the subjective valence of emotional pictures, but not to the self-relatedeness of the same stimuli, which, however, did modulate the activity of MPFC (Phan et al., 2004). It has to be noted, however, that the two dimensions of self-relatedeness and self-reference are not clearly distinguished at both the conceptual and experimental levels. Moreover, the self-relatedeness task activated regions that are well known to be responsible for self-referential processing (e.g., self-representation, semantic and episodic autobiographical memory retrieval; Martinelli et al., 2013). Overall, the functional similarity of neuronal populations in the amygdala and the OFC and their strong reciprocal connectivity support the view that these two regions are pivotal for coding the state value of an event (Salzman et al., 2007; Morrison and Salzman, 2009; Salzman and Fusi, 2010).

Taken together, these findings show that the amygdala responds to stimuli whose relevance for the organism is contextually and cognitively modulated, regardless of their valence (positivity and negativity) and beyond their social dimension.

## AN EVOLUTIONARY PSYCHOLOGICAL THEORY OF THE AMYGDALA

As proposed by Brothers (1990), the amygdala, together with the OFC and the STG, is part of a network of neural regions that constitutes the “social brain.” According to the social brain hypothesis (Dunbar, 2009), the size of the neocortex, which is mainly responsible for the expansion of the primate brain, has been found to be positively correlated with the increased complexity of social groups. Information-processing demands increase with the number of relationships as well as with the need to flexibly respond to the more complex scenarios of daily life. Within a large group, social interaction requires continuous on-line processing and monitoring of the dynamically and rapidly changing dispositions and intentions of conspecifics, as revealed by their bodily postures, facial expressions, or kinematics, and requires integration of this information with knowledge about their past actions, identity, and other social attributes.

Using a comparative method designed to detect coordinated evolution, Barton and Aggleton (2000) found that the amygdala and the neocortex volume correlated more strongly with each other, suggesting that these two distinct structures were conjointly tuned by natural selection to respond adaptively to particular lifestyles. Overall, the architecture of the prefrontal cortex is such that, on average, inputs from the amygdala attain approximately 90% of the prefrontal areas (Emery et al., 1997). Previous studies had already shown that between species amygdala volume was correlated with group size and the complexity of social networks. Cross-species comparative findings in non-human primates suggested that, when group size is taken as a proxy measure of social complexity, a significant positive correlation was found in 44 primate species between the relative amygdala volume (the ratio is estimated from total brain volume), and social group size, suggesting that this structure and in particular the *basolateral* nuclei, have evolved under evolutionary selectional pressure to increase the ability to manipulate information necessary to subserve sophisticated social relationships (Emery et al., 1997). More recently, Barger et al. (2007) also reported that larger amygdala, in particular the corticobasolateral complex, conjointly expanded with evolutionarily newer cortex under the pressure of the increased processing demands required by a complex social life. It has recently been shown that interindividual variability, both in humans (Bickart et al., 2010, 2012) and in primates (Sallet et al., 2011), is also linked with these parameters. Indeed, Bickart et al. (2010, 2012) showed that amygdala volume positively correlates with increasing network size and complexity (Bickart et al., 2010) and that stronger amygdala connectivity with other structures belonging to the social brain, such as the vMPFC, predicted group size and complexity. Importantly, this relationship was specific to the amygdala network and was not reported for other large scale functional networks, when controlling for age and correcting for multiple comparisons. Sallet et al. (2011) randomly assigned adult macaques to small or large social group housing conditions and found that several regions comprising the amygdala showed increased volume in the large social group. Taken together these findings suggest that interindividual differences in amygdala volume are strictly linked to social group size and complexity. Moreover, this variability seems sensible to environmental conditions and flexible to change even in adulthood. Even if no firm conclusion can be derived so far, the results of Sallet et al. (2011) suggest that reduced amygdala volume could be the consequence rather than the cause of individual social behavior. Although brain volume is an index of information-processing capacity, the fact that these two separate structures show closely correlated evolutionary changes in size reveals an increase in neural connectivity between them, in particular between the basolateral nuclei and the STG, the OFC and MPFC^2^.

Therefore, converging evidence suggests that the amygdala and the frontal cortex underwent expansion and evolved together by increasing neural connectivity. As we discussed above, from the perspective of the evolutionary psychology, the amygdala whose primary modular function was to rapidly and efficiently evaluate the environment for potentially threatening events (Amaral and Price, 1984; Öhman and Mineka, 2001; LeDoux, 2005) was constructed and adjusted in response to the statistical composite of situations encountered by our species in ancestral environments. However, because of such increased connectivity in the fronto-limbic neural circuit strongly characterizing the development of the human brain, the amygdala broadened its domain of specificity and enhanced the system’s ability to regulate and generate more flexible and adaptive social behavior.

The same neural system is rarely capable of solving different adaptive problems fast and efficiently since different information-processing systems usually instantiate distinct procedures for their successful solution (Cosmides and Tooby, 1994). What we argue here is that the amygdaloid complex has preserved the primitive function of self-relevance detector by reshaping its internal modular structure, likely by weakening some of its modular properties (e.g., limited central accessibility and informational encapsulation^3^), to flexibly respond to a larger variety of self-relevant evolutionarily unprecedented circumstances.

## THE RELEVANCE DETECTOR THEORY OF AUTISM

In the following, we argue that the complex pattern of emotional and socio-behavioral impairments typically reported in individuals with ASDs reflects a disruption of the neural system devoted to the processing of self-relevant information, primarily relying on the functional and connectivity integrity of the fronto-amygdala circuit. Indeed, as we discussed above, although the amygdala can process relevant stimuli in a reflexive and unconscious manner (Vuilleumier et al., 2003; Juruena et al., 2010), it serves the function of bringing to consciousness awareness self-relevant information through the mechanism of emotional arousal (Vuilleumier and Schwartz, 2001). Thus, a disruption of the Relevance Detector System would lead to an impairment in the conscious appraisal of self-relevance emotions, which would compromise the ability to represent and communicate one’s own internal states and feelings and lead to a reduce affective flexibility and emotional control (Cunningham et al., 2008).

A previous study on electrical stimulation suggested that the limbic system has a special role in bringing experience to a conscious level by associating affective and motivational significance with sensory information (Gloor et al., 1982). Neurobiological research has revealed that the neural substrates of self-awareness and subjective experience critically include the medial frontal cortex and the insula, both of which structures are functionally interconnected with the amygdala (Damasio, 1999; LeDoux, 2007). More recently, converging evidence from two studies (Kennedy and Courchesne, 2008; Lombardo et al., 2009) points to functional abnormalities in the vMPFC associated with self-related evaluative processing.

Research focused on emotional dysfunctions and theoretical accounts have emphasized the notion that the mechanisms mediating the self-regulation of behavior during social–emotional exchanges are severely impaired in ASDs (Yirmiya et al., 1992; Hobson, 1993). Pioneer studies reported that difficulties in children with ASDs might arise with both basic emotions (fear, disgust, anger) and social cognitive emotions (pride, embarrassment, shame) that are related to introspection and self-reflection (Capps et al., 1995; Loveland et al., 1997; Kasari et al., 2001; Heerey et al., 2003). It has been shown that children with autism have a less coherent representation of their own emotional experiences and failure to distinguish emotional experiences stems from a lack of reflective appraisal of those experiences (Harris et al., 1987). Despite preserved physiological responses and emotional empathy, they might often fail to show cognitive empathy (Rogers et al., 2007) or to generate and regulate emotionally laden situations introspectively (Rieffe et al., 2007). Recently, a consistent amount of evidence has pointed out that there is considerable overlap in the clinical presentation of persons with a diagnosis of ASD or of alexithymia (Hill et al., 2004; Hill and Berthoz, 2006), since both are characterized by disturbances in recognizing emotions and in the ability to use feelings to regulate interpersonal exchanges (Fitzgerald and Bellgrove, 2006). Remarkably, alexithymia can be regarded as a disrupted interaction between emotional arousal and the subjective experience of feelings (see Gaigg, 2012).

Different lines of behavioral research have reported disturbance in processing self-related information in individuals with ASDs, in terms of monitoring self-performed actions (Russell and Jarrold, 1999), or in correctly deciding whether an action had been produced by oneself or another agent (Russell and Jarrold, 1999). Millward et al. (2000) reported that children with autism have a specific difficulty with the recall of personally experienced events, as compared with memory for events experienced by a peer. Using a recognition test, Toichi et al. (2002) showed that a group of adults with HFA does not benefit from the self-reference effect since they are impaired in processing words in a self-related manner, in the absence of semantic and phonological impairments. More recently, Hare et al. (2007) found that adults with ASD demonstrate superiority for self-experienced events over events merely observed when the recall is cued whilst this superiority effect disappeared in free recall. On the same line, Zalla et al. (2010) have reported that adults with AS exhibited a reduced enactment effect for self-performed actions in free recall, as compared to a matched control group.

Although an abundant body of neuroimaging studies have related amygdala activation to the social dimension of stimuli (i.e., eye contact, gaze orientation, biological actions and intentions, trustful faces), the *Relevance Detection Theory of Autism* predicts that the amygdala specifically responds to self-relevant information. The literature is, however, extremely varied with respect to the physiological responses to socio-emotional events, associated with amygdala functionality in ASDs. In fact, ASD individuals are found to be either hyper- or hypo-aroused in response to simple sensory stimuli, or stimuli varying in emotional valence or social dimensions (Dalton et al., 2005; Schultz, 2005; Schoen et al., 2008; Anderson and Colombo, 2009; Ball et al., 2009). The *Relevance Detection Theory of Autism* posits that hyper-activation of the amygdala in response to potentially threatening or physically intense events in the environment is due to a disrupted interplay between a cognitive “top-down” attentional system and an automatic “bottom-up” attentional mechanism operating on raw sensory input. Since the amygdala automatically and rapidly detects salient physical and biological features of potential importance by enhancing *bottom-up* attentional resources, reduced effective top-down control and attentional modulation exercised by the vMPFC on this structure would lead to the inability to form a “priority map of saliencies” that allows to regulate behavior and navigate the complex social world. Thus, in this view, reduced eye contact and social withdrawal are the result of adaptive avoidance responses to overcome excessive stimulation by a physically intense world or emotional hyperarousal and overresponsiveness to potentially aversive events (see Dalton et al., 2005; Kylliäinen and Hietanen, 2006; Kliemann et al., 2010).

Among the social stimuli, the eyes constitute an special source of relevant information. The ability to discriminate eye direction is thought to reflect an innate predisposition and a primitive function (Scaife, 1976). For many of species, direct gaze generally signals hostility and threat, and is associated with escape behavior (Emery et al., 1997, 2001). In monkeys, perceived eye gaze contact is associated with amygdala activation (Emery et al., 1997). In humans, eye gaze is a salient stimulus constituting an important source of information about other conspecifics (e.g., identity, age, gender, mental states, and internal emotional dispositions) but, depending on cultural and context-related factors, mutual eye contact and direct gaze may not necessarily be intrinsically threatening. Thus, because these signals can be ambiguous, their decoding may necessitate additional cognitive information and more conscious, evaluative processes (Engelmann and Pogosyan, 2013). The disrupted functionality of this integrative *Relevance Detection System* might lead to abnormal sustained activation of this subcortical route and to failure to detect meaningful aspects of the environment, in accordance of a “priority map” integrating intrinsic and extrinsic salience stimuli.

This explanation is in accordance with previous studies showing altered functional connectivity between vMPFC and amygdala, associated with diminished habituation of amygdala response to emotional faces (Swartz et al., 2013). Intriguingly, South et al. (2008) have shown that individuals with ASD exhibit a “threat advantage” effect (faster response time in detection of threatening stimuli as compared to neutral ones) and a typical anger superiority effect in visual search tasks employing face stimuli. In a recent neuroimaging study (Dalton et al., 2005), the amount of eye gaze fixation was strongly correlated with amygdala activation when viewing both emotional and neutral faces in participants with ASD, but not in control participants.

According to Liddell et al. (2005), an “innate alarm system,” mediated by the primitive subcortical pathway, enables the organism to detect potentially threatening stimuli or unpredictable events in the physical environment, and thus promotes withdrawal and escape behaviors. In typically developed individuals, automatic fear-driven amygdalar responses are followed by activation in brain areas associated with controlled and reflective processes (Liddell et al., 2005). Indeed, amygdalar abnormalities typically associated with difficulties with fear extinction (Davis, 1992, 2000), also involve disturbances in social anxiety, hyperarousal, and sensory over-responsivity in ASDs (Amaral et al., 2008; Green and Ben-Sasson, 2010). Increased amygdala volume in children with ASDs was found to be positively correlated with anxiety and severity of social-communication deficits (Amaral et al., 2008) and higher scores for social anxiety show greater right amygdala response to negative emotional expressions in participants with ASDs (Kleinhans et al., 2010). While Mogg and Bradley (1999) regarded anxiety as preattentive bias toward threat, and argued that it results from an automatic encoding of threat without modulatory and elaborative processing, according to Davis and Whalen (2001), pathological anxiety may not be a disorder of fear, but a deficit in the ability to regulate vigilance and generalized hyperarousal in response to potential threat.

It is likely that reduced eye contact, perceived as potentially aversive stimuli, would preclude the development of perceptual expertise for faces, and hamper the ability to process different types of self-relevant social information acquired through faces, such as emotions, intentions, and trustworthiness (Begeer et al., 2008; Harms et al., 2010), and thereby trigger a cascade of deficits in this population in the domain of social interaction, such as initiated joint attention (Mundy and Newell, 2007), communication and attachment behavior (Hobson, 1993; Davies, 1994; Hobson and Lee, 1998).

Interestingly, the administration of oxytocin, a neuropeptide, which is known to be lower in individuals with autism (Modahl et al., 1998), enhances the salience and retention of social information in individuals with autism (Hollander et al., 2007; Andari et al., 2010) and decreases repetitive behaviors (Hollander et al., 2003). Recently, in a neuroimaging study, Domes et al. (2013) found that the oxytocin treatment promotes face processing and eye contact in individuals with ASDs and increases right amygdala activity. The medial nucleus of the amygdala, through the actions of the oxytocin, is a critical site for regulating approach and avoidance behaviors, for promoting social attachment (Ferguson et al., 2001), and for reducing anxiety (Bartz and Hollander, 2006).

Beyond the socio-emotional domains, our theory predicts that a disrupted functionality of this integrative *Relevance Detection System* might lead to the abnormal capture of attention by low-level, bottom-up visual properties of the stimuli (e.g., intensity, color, contrast, orientation), due to enhanced sensitivity of the physical attributes of the stimulus (Joseph et al., 2009). While this hyper-sensitivity is often associated with superior visual search abilities in ASDs, the enhancement of low-level visual processing and of physical salience of the events might lead to allocation of attention to irrelevant aspects of the visual environment (Joseph et al., 2009; Kaldy et al., 2011).

The hyper-sensitivity to the physical salience of external stimulation would lead to failure to shape a valid priority map of saliencies which could allow sensory stimuli to be integrated with current goals, personal needs, and contextual and prior knowledge. While bottom-up attention is driven by visually salient events in the environment (Itti and Koch, 2001), top-down attentional mechanisms implement longer-term cognitive strategies, biasing attention toward salient features as a function of the organism’s internal needs and goals (Connor et al., 2004). This hypothesis is in accordance with recent findings showing that individuals with ASDs require a higher signal-to-noise ratio for the discrimination of visual or auditory presentations of fear *vs* disgust expressions (Charbonneau et al., 2013). Recently, Amso et al. (2013) found that, relative to control children, children with ASDs rely more on bottom-up physical information for initial attention guidance, despite a similar orienting to faces in the two groups. Importantly, this finding suggests that reduced attention to faces and gaze in ASDs does not reflect disruption of an innate system devoted to the detection of eye contact, nor the lack of social motivation, but it would be the result of an unbalanced reliance on physical features of the environment. As also posited by the “Intense World Theory” (Markram et al., 2007), the hyper-emotionality, reflecting hyper-functionality of the limbic system, together with excessive responsiveness to environmental stimulation, result in perception of an aversive world, and social withdrawal in individuals with ASDs.

Taking into account the distinction we make between intrinsic and extrinsic context-dependent salience of the stimulus, a possible operationalization in an experimental setting would be to orthogonally manipulate these parameters to test which specific aspect of salience detection is impaired in people with ASDs and the corresponding response in the amygdala. Based on our current knowledge, we hypothesize that participants with ASDs would be more responsive to the bottom-up physically salient features associated with prolonged amygdalar activity, while diminished impact of the contextual contingency (extrinsic salience) may reflect reduced modulatory affect exercised by prefrontal regions on amygdala activity.

## CONCLUSION

In the present review, we have proposed that an early emerging neurological insult to the interconnected fronto-amygdala circuit disrupting the ability to flexibly and adaptively orient attention toward self-relevant stimuli might be a primary deficit of ASDs. Specifically, the amygdala is responsible, in concert with the vMPFC, of the formation of a *priority map* of self-relevant events that might be accessible to and modulated by conscious evaluative processes. This priority map includes stimuli whose salience is determined by their intrinsic biological significance, the physical properties or the extrinsic contextual situation. In this view, physically intense stimulation and emotionally arousing events, associated with the amygdala hyperactivation, are actively avoided thus producing reduced attendance to meaningful aspects of the environment, including the social ones, and deficits in the self-regulation of behavior. At the neural level, our theory is in accordance with the fronto-amygdala disconnectivity explanation and the hyper-active models which posit that the amygdala hyperactivation results from a defective top-down modulation by prefrontal areas involved in conscious evaluative processes. Moving away from the classical account of the amygdala as a *threat detector* or a socio-emotional processing submodule would favor the design of studies that might provide the opportunity to account for heterogeneities of cognitive phenotype and symptomatology across the autistic spectrum.

## Conflict of Interest Statement

The authors declare that the research was conducted in the absence of any commercial or financial relationships that could be construed as a potential conflict of interest.

## Footnotes

1 This subdivision, originally proposed by Amunts et al. (2005), *using* post-mortem cytoarchitectonic mapping, was recently replicated *in vivo* using diffusion tensor imaging (Solano-Castiella et al., 2010; Bach et al., 2011), high field structural MRI (Solano-Castiella et al., 2011), functional connectivity-based parcellation (Mishra et al., 2013), and meta-analytic procedures (Bzdok et al., 2012) reporting either two- or three-cluster solutions.

2 Besides the well-recognized connections with medial and orbital regions of prefrontal cortex, the amygdala is also connected to the lateral prefrontal cortex, albeit to a lesser degree.

3 According to Fodor (1983), a system is informationally encapsulated when the information is processed in a purely feedforward (bottom-up) manner: it is not affected by the person’s expectations, beliefs, or desires and it is inaccessible to consciousness, and hence unavailable for explicit report. Limited central accessibility is a closely related property which involves restriction on the flow of information out of the system.
